# *Schistosoma mansoni* infection and undernutrition among school age children in Fincha’a sugar estate, rural part of West Ethiopia

**DOI:** 10.1186/1756-0500-7-763

**Published:** 2014-10-27

**Authors:** Zeleke Mekonnen, Selima Meka, Ahmed Zeynudin, Sultan Suleman

**Affiliations:** Department of Medical Laboratory Sciences and Pathology, College of Public Health and Medical Sciences, Jimma University, P.O. Box 378, Jimma, Ethiopia; Department of Pharmacy, College of Public Health and Medical Sciences, Jimma University, P.O. Box 378, Jimma, Ethiopia

**Keywords:** *Schistosoma mansoni*, Under nutrition, Stunting, Wasting, School children, Ethiopia

## Abstract

**Background:**

Parasitic infection like schistosomiasis is known to exert a negative effect on nutritional status of school-aged children. However, studies associating parasitic infections with undernutrition are scarce. Thus, this study was primarily to document the association between *Schistosoma mansoni* infection and undernutrition among school-aged children in a rural setting of Fincha’a Sugar Estate, Ethiopia.

**Methods:**

A cross-sectional study was conducted on a total of 453 school-aged children (5-18 years). Stool specimen was collected and examined using the standard Kato-katz technique. Children’s height-for-Age Z-score (HAZ) and Body mass index-for-Age Z- score (BAZ) was determined. Z-Scores for each nutritional index were compared with the WHO child growth standards reference values. Children were considered stunted or wasted as HAZ or BAZ falls below -2 standard deviations, respectively.

**Result:**

The overall prevalence of *Schistosoma mansoni* infection was 53.2%. Out of the total school children examined, 11.5% and 13.2% were stunted and wasted, respectively. Multivariate logistic regression analysis was done to determine the relationship between S*chistosoma mansoni* infection and nutritional status controlling for other factors. Accordingly, stunting was not significantly associated while wasting was negatively associated with S*chistosoma mansoni* infection. Paternal occupation was the best predictor of stunting and wasting such that, unemployed fathers have 4.28 (95% CI; 2.13, 8.63) (p < 0.001) and 3.83, 95% CI; 1.89, 7.79) (p < 0.001) chance of having stunted and wasted children, respectively.

**Conclusion:**

*Schistosoma mansoni* infection is highly prevalent in the study area. The high prevalence of wasting, and moderate level of stunting among study subjects in this study area indicate that they are affected by both infection and undernutrition. Therefore, regular preventive chemotherapy against *S. mansoni* and other control measures are recommended. Moreover, possibilities of synchronized nutritional rehabilitation and creation of employment opportunities to the families should be looked for.

## Background

In the past, nutrition and health of school-aged children and adolescents in the developing world received little attention relative to those less than five years of age. However, there is increasing evidence, resulting in international concern, that the high level of nutritional deprivation combined with the heavy burden of disease in school age group has negative consequences for a child’s long term overall development. This has prompted an increased focus on the diverse needs of the school-aged child [1–3].

Recent studies on school-aged children have shed new information on stunting, wasting and underweight for this age group [4–6]. Currently, about one-fourth of African primary school children lie under the fifth centile of United States- National Center for Health Statistics (US-NCHS) reference for Height-for-Age Z-score (HAZ). While undernutrition prevalence has decreased significantly in most other developing countries in the last few years, it has been nearly static for sub-Sahara Africa [7].

While factors affecting growth and development of preschool children are well-documented, the factors influencing growth of school-aged children are not as such clearly elucidated [8, 9]. One of the factors emphasized in the 1993 World Bank report was the relationship between parasitic infection and undernutrition [10]. Moreover, the relationship between undernutrition and soil transmitted helminth infection has been well established [11–13], but such studies have been limited only to soil transmitted helminth infections in children [14–17].

However, the relationship between undernutrition and schistosomiasis is now gaining more attention. There are few studies which associate the different species of schistosoma with linear growth retardation in children [5, 18, 19]. This parasitic infection is known to exert a negative effect on the nutritional status of school-aged children even at a low to moderate intensity of infection [20, 21]. Such studies are limited in developing countries like Ethiopia, where schistosomiasis is endemic. The aim of this study was, therefore, to investigate the association between *S. mansoni* infections and prevalence levels of undernutrition, and related associated risk factors influencing this relationship among school-aged children in a rural setting of Fincha’a Sugar Estate, Western Ethiopia.

## Methods

### Study area and population

A cross-sectional study was conducted from February - March, 2011 among school-aged children (5-18 years) residing in a rural village of Fincha’a Sugar Estate in Horo Guduru Wollega, western Ethiopia. Fincha’a Sugar Estate is found in Oromia regional government and located at 350 km west of Addis Ababa (Capital of Ethiopia). Fincha’a valley is bounded by escarpments from east, west and south situated at a latitude of 9° 30′N to 10° 60′N and a longitude of 37° 15′ to 37° 30′E. The area is characterized by a semi-arid type climate having the mean maximum and mean minimum temperatures of 30.6°C and 14.5°C, respectively. It is situated 1,350 m -1,600 m above sea level with mean relative humidity of 62% and receiving mean annual precipitation of 1,485 mm. The population comprises of different ethnic groups that settled at different times in search of job opportunities. The total population of the area is estimated to be over 18,000 according to the 2007 population census, with male/female ratio of approximately 1.1:1.0 [22]. All kind of basic health services were provided by one health center and 5 small clinics to all communities residing in the sugar farm (camp) across the different villages.

**Figure 1:**
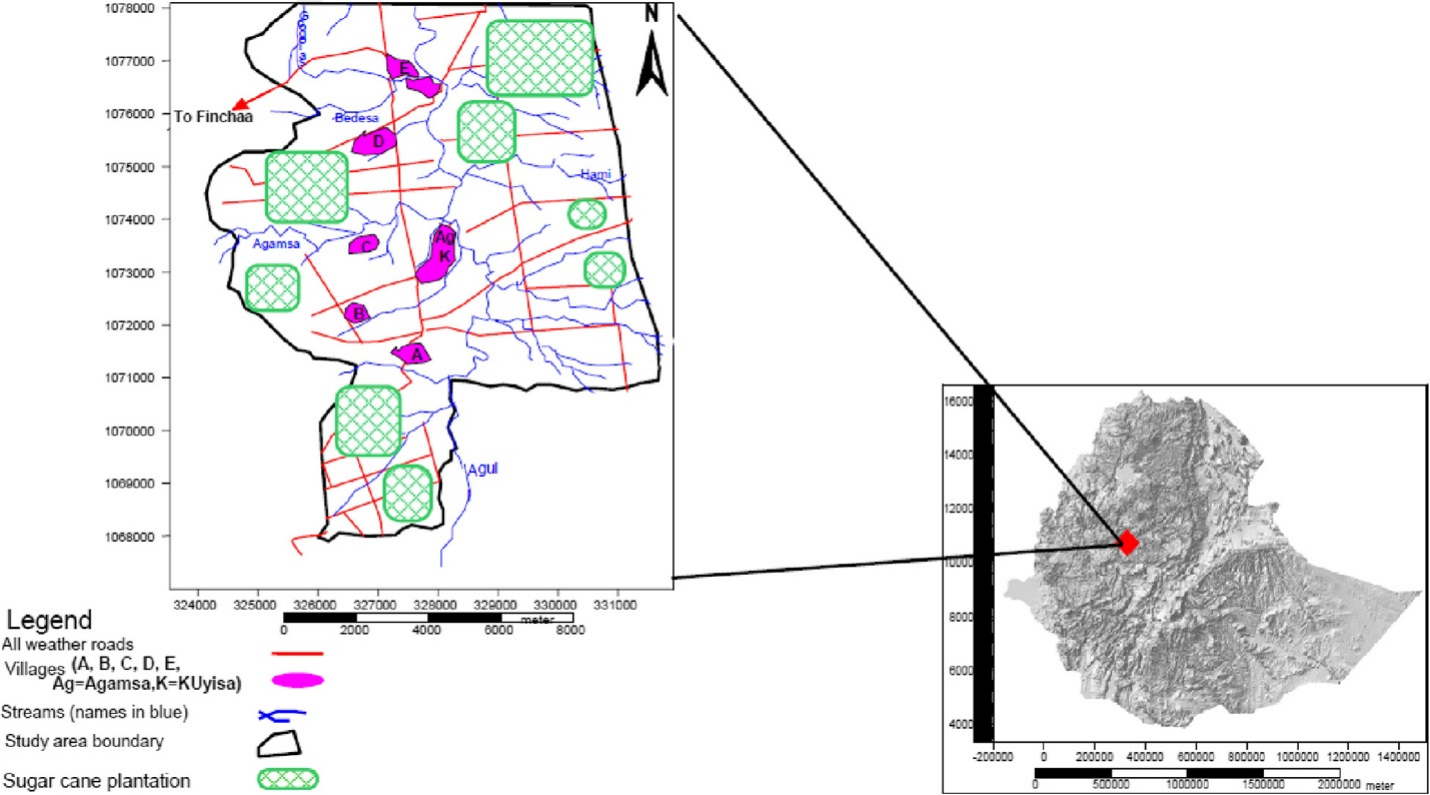
**Map of study villages within Fincha’a sugar factory, West Ethiopia.** [*Source*: MSc document (Bayissa Chala), AAU, 2007].

The Fincha’a Sugar Estate was selected purposively as the area is a focus of *S. mansoni* infections due to its favorable environment (sugarcane cultivation and availability of a special watershed of the hydropower dam reservoir). The sugar factory was organized into 5 farm villages (Village A, B, C, D, and E) and two small towns (Agamsa and Kuyisa). From a total of 5 villages in the area, two (village ‘A’ and ‘E’) were selected using lottery method for this study (Figure 1). The sample size was determined assuming a confidence interval of 95% and a 60% prevalence of *S. mansoni* in the study area. A sample size of 458 was considered to be adequate, of which 453 students (242 from village ‘A’ and 211 from village ‘E’) participated after the sample size was proportionally distributed over the two villages based on the total households in each of the two villages. Then, one child from each randomly selected household was recruited using simple random sampling technique for inclusion in the study. The variables used for inclusions were age range of 5-18 years, lived primarily in the study village at least for the last two years, provided both child assent and parental consent and being asymptomatic for other diseases at the time of data collection.

### Socio-economic data

Data on socio-demographic data such as age, sex, parental and maternal educational status and occupational status was obtained by interviewing parents and legal guardians guided by pre-tested structured questionnaire. Data on socio-economic status addressing household durable assets (radio, television, bicycle, mobile phone etc), three components of dwelling structure (roof, floor and wall type) and number of rooms for dwelling were assessed. Questions dealing with household asset index were adopted from Ethiopia demographic and health survey 2005 [23]. A household wealth index was constructed from 20 variables using principal component analysis. Commonly used arbitrary cut-off points were applied to classify children as the lowest 40% of the socio-economic status score into ‘poor’ , the highest 20% as ‘rich’ and the rest as the ‘middle’ group [24].

### Stool examination

Standard Kato-katz cellophane faecal thick smear method (41.7 mg template) was utilized to examine three consecutive days stool specimen for the presence of *Schistosoma mansoni, Ascaris lumbricoides, Trichuris trichiura* and hookworm infection. Microscopic examination was done within 30-60 min of smear preparation not to miss hookworm infection since the sensitivity of Kato-katz techniques to detect hookworm decreases after an hour [25]. The mean intensity of infection was expressed as eggs per gram (epg) and classified according to World Health Organization (WHO) criteria as low (1-99 epg), moderate (100-399 epg), and heavy (≥400 epg) intensity infection [26].

### Anthropometric measures

Body weight with light clothing was measured to the nearest 0.1 kg on a Seca Model 880 Digital scale (Hanover, MD, Germany). Height was measured barefooted to the nearest 0.1 cm using a portable stadiometer that was taped to a wall at the same location for each measurement. Height-for-Age Z-score (HAZ) and Body mass index for-Age Z-score (BAZ) were based on the WHO child growth standards. Body mass index (BMI) is the index of choice for the assessment of recent under-nutrition in both adults and adolescents [27]. Stunting was defined as HAZ < -2 standard deviations (SDs) and wasting or wasted if BAZ < -2 SDs compared with a normal, healthy reference population. As part of data quality assurance, in addition to test-retest and inter-rater reliability assessments, all anthropometric measurements were taken with calibrated and validated instruments.

Age of each participant was collected from the mother and counter checked using vaccination cards, baptismal certificates or other forms of informal recording. When these recordings were not available, a calendar of locally important events was used. The 15^th^ day of the month was used when the date of birth is unknown and if the month of birth is unknown, the midpoint of the year of birth was used.

### Data processing

Data were coded and entered into SPSS for windows version 17 statistical software package for analysis of variables after being checked for completeness and cleaned of any inconsistencies. Simple frequency and percentages were used as the statistical parameter in the descriptive analysis. Bivariate logistic regression was used to identify predictors for under-nutrition. Multivariate logistic regression analysis was used to provide independent estimates of the relationships of interest. P <0.05 was considered significant.

### Ethical consideration

Ethical clearance was obtained from Ethical Clearance Committee of Jimma University. The study participants involved in the study after informed written consent was obtained from their parents or guardians and assent from participating children. Children positive for *S. mansoni* have received single dose of praziquantel (Biltricide®, Bayer AG, Germany), 40 mg/kg body weight according to standard treatment guideline of Ethiopia (2010) and study participants identified as stunted and wasted were referred to Fincha’a sugar estate health center for necessary interventions.

## Results

A total of 458 children between 5-18 years of age were recruited in the study. Complete parasitological results were available for 453 (98.9%), as 5 were withdrawn since they were unfit to the inclusion criteria. Out of the total study participants 216 (47.7%) were males and 237 (52.3%) were females. Most of the study participants (62.7%) belongs to the age group 10-14 followed by 25.6% in the age group 5-9 (Table 1). The mean HAZ and BAZ were -0.69 ± 1.13 SD and -0.83 ± 0.96 SD, respectively. The overall prevalence of stunting was 11.5% and 13.2% were wasted. The overall prevalence of *S. mansoni* infection was 53.2% with a mean ± SD intensity of 358 ± 477 epg. The infection intensity was light in 21.4%, moderate in 15.7% and heavy in 16.1% of the participants. Soil transmitted helminthes were not detected in any of the study participants.

**Table 1:** **Socio-demographic characteristics of school age children, Fincha’a Sugar Estate, Horo Guduru Wollega, Ethiopia, Feb-Mar, 2011**

Socio-demographic variables		Frequency	Total
		n (%)	n (%)
**Gender**	Male	216 (47.7)	453 (100)
	Female	237 (52.3)	
**Age group**	5-9	116 (25.6)	453 (100)
	10-14	284 (62.7)	
	15-18	53 (11.7)	
**Maternal literacy**	Illiterate	338 (74.6)	453 (100)
	Literate	115 (25.4)	
**Paternal literacy**	Illiterate	321 (70.8)	453 (100)
	Literate	132 (29.2)	
**Maternal occupation**	Unemployed	395 (87.2)	453 (100)
	Employed	58 (12.8)	
**Paternal occupation**	Unemployed	48 (10.6)	453 (100)
	Employed	405 (89.4)	
**Total**			**453 (100)**

### Stunting and *Schistosoma mansoni*infection

Bivariate logistic regression analysis showed that, stunting was not associated with *S. mansoni* infection. However, it was related with mother’s literacy and father’s occupational status. Accordingly, children born from illiterate mothers were about 3 times (95% CI; 1.19, 6.89) more likely to be stunted (p = 0.02) than their counterparts. Children of unemployed fathers had about 5 times (95% CI; 2.49, 9.86) higher risk of being stunted (p <0.001) than children of employed fathers (Table 2).

**Table 2:** **Bivariate and multivariate logistic regression analysis of factors associated with stunting amongst school age children residing in Fincha’a Sugar Estate, Horo Guduru Wollega, Ethiopia, Feb-Mar, 2011**

Variables	Stunted	Normal	COR	P-value	AOR	P-value
	n (%)	n (%)	(95% CI)		(95% CI)	
**Gender**						
Female	23 (9.7)	214 (90.3)	1.00		1.00	
Male	29 (13.4)	187 (86.6)	1.44 (0.81, 2.58)	0.22	1.25 (0.69, 2.29)	0.46
**Age group**						
5 – 9	12 (10.3)	104 (89.7)	1		-	
10 – 14	30 (10.6)	254 (89.4)	1.02 (0.51, 2.08)	0.95	-	
15 – 18	10 (18.9)	43 (81.1)	2.02 (0.81, 5.01)	0.13	-	
***S. mansoni***						
Uninfected	26 (12.3)	186 (87.7)	1.00		-	
Infected	26 (10.8)	215 (89.2)	0.86 (0.48, 1.54)	0.62	-	
**SES Index**						
Poor	21 (11.6)	160 (88.4)	1.00		-	
Middle	23 (12.7)	158 (87.3)	1.12 (0.59, 2.09)	0.75	-	
Rich	8 (8.8)	83 (91.2)	0.73 (0.31, 1.73)	0.48	-	
**Maternal literacy**						
Literate	6 (5.2)	109 (94.8)	1.00		1.00	
Illiterate	46 (13.6)	292 (86.4)	2.86 (1.19, 6.89)	0.02	2.30 (0.94, 5.63)	0.07
**Paternal literacy**						
Literate	16 (12.0)	117 (88.0)	1.00		-	
Illiterate	36 (11.2)	284 (88.8)	0.93 (0.49, 1.74)	0.81	-	
**Maternal occupation**						
Employed	5 (8.6)	53 (91.4)	1.00		-	
Unemployed	47 (11.9)	348 (88.1)	1.43 (0.55, 3.76)	0.47	-	
**Paternal occupation**						
Employed	37 (9.1)	368 (90.9)	1.00		1.00	
Unemployed	15 (31.2)	33 (68.8)	4.96 (2.49, 9.86)	<0.001	4.28 (2.13, 8.63)	< 0.001
**Total**	**52**	**401**				

Multivariate logistic regression analysis was conducted to fit into a model including all variables with a *P*-value <0.25 in the bivariate analysis for stunting. Therefore, gender, age group of the study participants, mother’s educational status and paternal occupation were included in the model for analysis. Controlling for other factors, paternal employment was the best predictor of stunting among school-age children such that, unemployed fathers have about 4 times (95% CI; 2.13, 8.63) more chance of having stunted children (p < 0.001) (Table 2).

### Wasting and *Schistosoma mansoni*infection

Wasting was significantly associated with *S. mansoni* infection. Overall, S. mansoni infected children were 0.45 times (95% CI; 0.25, 0.81) less likely to be wasted (Table 3), where as children with light infection (21.4%) were about 2 times less likely to be wasted than uninfected children (p = 0.01). Wasting was also associated with gender, maternal literacy and paternal occupation. Males were 2.69 times (95% CI; 1.51, 4.79) more likely to be wasted than their female counters (p = 0.001). Children born from illiterate mothers were about 4 times (95% CI; 1.67, 10.96) more likely to be wasted than children born from literate mothers (p = 0.002). Likewise, children born from unemployed fathers were about 4 times (95% CI; 2.29, 8.69) more likely to be wasted than their counter parts (p <0.0001). Controlling for other factors, the AOR indicates that father’s employment status is the best predictor of wasting among school age children (AOR = 3.83, 95% CI; 1.89, 7.79) (p < 0.001).

**Table 3:** **Bivariate and multivariate logistic regression analysis of factors associated with wasting amongst school age children residing in Fincha’a Sugar Estate, Horo Guduru Wollega, Ethiopia, Feb-Mar, 2011**

Variables	Wasted	Normal	COR	P-value	AOR	P-value
	n (%)	n (%)	(95% CI)		(95% CI)	
**Gender**						
Female	19 (8.0)	218 (91.0)	1.00		1.00	
Male	41 (19.0)	175 (81.0)	2.69 (1.51, 4.79)	0.001	2.18 (1.19, 3.96)	0.01
**Age group**						
5 – 9	11 (9.5)	105 (90.5)	1.00		-	
10 – 14	38 (13.4)	246 (86.6)	1.29 (0.65, 2.59)	0.46	-	
15 – 18	11 (20.7)	42 (79.3)	2.27 (0.93, 5.55)	0.07	-	
***S. mansoni***						
Uninfected	38 (17.9)	174 (82.1)	1.00		1.00	
Infected	22 (9.1)	219 (90.9)	0.46 (0.26, 0.81)	0.01	0.45 (0.25, 0.81)	0.00
**SES Index**						
Poor	25 (13.8)	156 (86.2)	1.00		-	
Middle	20 (11.0)	161 (89.0)	0.78 (0.41, 1.45)	0.43	-	
Rich	15 (16.5)	76 (83.5)	1.23 (0.61, 2.47)	0.56	-	
**Maternal Literacy**						
Literate	5 (4.3)	110 (95.7)	1.00		1.00	
Illiterate	55 (16.3)	283 (83.7)	4.28 (1.67, 10.96)	0.002	3.56 (1.36, 9.30)	0.01
**Paternal literacy**						
Literate	15 (11.3)	118 (88.7)	1.00		-	
Illiterate	45 (14.2)	272 (85.8)	1.29 (0.69, 2.40)	0.43	-	
**Maternal occupation**						
Employed	5 (8.6)	53 (91.4)	1.00		-	
Unemployed	55 (13.9)	340 (86.1)	1.72 (0.66, 4.48)	0.27	-	
**Paternal occupation**						
Employed	43 (10.6)	361 (89.4)	1.00		1.00	
Unemployed	17 (34.7)	32 (65.3)	4.46 (2.29, 8.69)	0.00	3.83 (1.89, 7.79)	0.00
**Total**	**60**	**393**				

## Discussion

Undernutrition continues to be a major health burden in developing countries. Since parasitic infections cause anorexia and poor absorption of nutrients and promote the deviation of nutrients to the organism's defense mechanisms, they contribute to the onset or exacerbation of weight and height deficits [28, 29]. Previous cross-sectional studies have reported clear relationship between *S. mansoni* infection and nutritional status.

The mean HAZ and BAZ were below zero indicating that the distribution was shifted downwards [30]. The overall prevalence of stunted and wasted school-aged children in Fincha’a Sugar Estate was 11.5% which is considered low (<20%) according to the WHO classification [30]. Similarly, the prevalence of wasting was 13.2%, which is considered high (10-14%).

When these figures were compared with other study, stunting was almost 1/3 of the prevalence reported among school-aged children of Peru [31]. This could be attributed to the difference in transmission of geo-helminth in the two study areas. In our study, STH infection was not detected from any of the participants. However, 86% of the children from Peru were infected with at least one helminth and these may also have had an impact on nutritional status and contributed to high prevalence of stunting. Previously, it has been established that soil transmitted helminth infections are associated with stunting in pre-school and school children [32, 33]. The prevalence of wasting on the other hand was comparable with this study. This could be due to wasting being an indicator of acute nutritional insult might not be affected by intestinal parasites. This has been confirmed by significant negative relation between intestinal helminth infections and children’s growth in a study carried out in São Paulo, Brazil [17].

The prevalence of *S. mansoni* infection was 53.2% which is considered a high prevalence according to WHO classification of prevalence of *S. mansoni* [34]. Stunting was not statistically associated with *S. mansoni* infection. This was not the case in a previous cross-sectional study in the northeastern Brazilian town of Nazaré, Bahia [35]. In this study, children infected with *S. mansoni* were significantly more malnourished than children with a negative stool sample for several anthropometric variables. The reason for this contradicting finding could be due to difference in transmission of geo-helminthes. The study population in Brazil was highly infected with soil transmitted helminthes (STHs). The prevalence of *Ascaris lumbricoides* and *Trichuris trichiura* infections among those children were 71.8 and 67.9%, respectively. However, in our case none of the STHs were detected in stool examination. One major reason for the absence of STHs in our study area could be the regular administration of anti-helminthic (mass chemotherapy) to children at the beginning of every school year according the source of the Fincha’a health center authorities.

It has been reported that intestinal helminthiasis may play a contributory role in the occurrence of childhood malnutrition [29, 36, 37]. One reason why our reports contradicts with the finding of the Brazil study might be that in endemic areas where more than one species of parasite prevails and that the interaction of these parasites with each other may modulate or change the parasite nutritional status relationships. Hence, we presume that a synergistic effect of STH and *S. mansoni* infection could be accountable for the nutritional deficit. Furthermore, the nutritional status of our participants might have been boosted by subsequent treatments.

In contrary to stunting *S. mansoni* was associated with wasting. However, the relation was surprising that infected children were less likely to be wasted than their uninfected counterparts. Wasting indicates recent nutritional condition and is generally associated with recent illness and failure to gain weight or a loss of weight [38]. *S. mansoni* infection, on the other hand, is a chronic disease and it’s relation with undernutrition is better explained by stunting. Therefore, we assumed long-term linear growth retardation might have relative protection of weight and height based indices (BAZ). Similar scenario has been reported in Philippines [39].

Our results on socio-economic status showed that Father’s occupation was the best predictor of both stunting and wasting among school age children. Similar study conducted in India indicated that the poor nutritional status was associated with socioeconomic variables such as sex of the child and father’s occupation [40]. In contrast to this, the study conducted in Peru and Peruvian Andes showed that father’s engagement on any type of occupation did not have differential impact on child undernutrition [41].

Moreover, the results of the study indicated that there seemed a conflicting relationship between SES of no association and paternal employment as a predictor of stunting/wasting. Although, most of the time wealth index is taken as proxy to employment, this conflicting relationship could be justified as 1) paternal employment might not necessarily be reliable indicator for wealth index in the study area and 2) most of the time, people do not disclose genuine enough information on their SES.

Our finding of maternal literacy as an associated factor with wasting is in line with the findings of several other studies conducted in India [40], Pakistan [42] Bolivia [43] Kenya [44] and Jamaica [45] which indicated that uneducated mothers were likely to have malnourished children than those educated. In contrast to the above findings, study conducted by Mazumdar in Egypt did not find any significant relationship between long-run child undernutrition indicator (stunting) and maternal education [46]. However, as undernutrition is the product of the interaction of many factors and confounded by those factors [38], it is difficult to reach on conclusive comparison on similarity and differences among different studies including the present study.

### Limitation of the study

We acknowledge inaccuracy of the age of the included children (for some of our study subjects) due to lack of birth certificates and recall bias that can be considered as one of the limitation of this study. Moreover, we use only BMI which depends upon weight and the square of height, it ignores basic scaling laws whereby mass increases to the 3^rd^ power of linear dimensions. Lastly we admit the effect of the study design as well.

## Conclusion

The high prevalence of *S. mansoni* infection and wasting, moderate level of stunting, and observed significant association mean that school children in these schistosomiasis endemic areas were affected by both infection and undernutrition. Therefore, regular screening and provision of mass treatment (preventive chemotherapy) against *S. mansoni* infections with other control measures are recommended. Moreover, possibilities of synchronized nutritional rehabilitation and creation of employment opportunities to the family of the child should be looked for.

## Abbreviations

HAZ: Height-for-Age Z-score
BAZ: Body mass index for-Age Z-score
BMI: Body mass index
SD: Standard deviations
AOR: Adjusted odds ratio
epg: Eggs per gram.
